# Investigation on the Mechanical and Electrical Behavior of a Tuning Fork-Shaped Ionic Polymer Metal Composite Actuator with a Continuous Water Supply Mechanism

**DOI:** 10.3390/s16040433

**Published:** 2016-03-25

**Authors:** Guo-Hua Feng, Wei-Lun Huang

**Affiliations:** 1 Department of Mechanical Engineering, National Chung Cheng University, Chiayi 621, Taiwan; ben_77920@hotmail.com; 2 Advanced Institute of Manufacturing with High-Tech Innovations, National Chung Cheng University, Chiayi 621, Taiwan

**Keywords:** ionic polymer, composite, electrochemical model, faradaic current, cation

## Abstract

This paper presents an innovative tuning fork-shaped ionic polymer metal composite (IPMC) actuator. With an integrated soft strain gauge and water supply mechanism (WSM), the surface strain of the actuator can be sensed* in situ*, and providing a continuous water supply maintains the water content inside the IPMC for long-term operation in air. The actuator was fabricated using a micromachining technique and plated with a nickel electrode. The device performance was experimentally characterized and compared with an actuator without a WSM. A large displacement of 1.5 mm was achieved for a 6 mm-long prong with 7-V dc actuation applied for 30 s. The measured current was analyzed using an electrochemical model. The results revealed that the faradaic current plays a crucial role during operation, particularly after 10 s. The measured strain confirms both the bending and axial strain generation during the open-and-close motion of the actuator prongs. Most of the water loss during device operation was due to evaporation rather than hydrolysis. The constructed WSM effectively maintained the water content inside the IPMC for long-term continuous operation.

## 1. Introduction

Recently researches on electroactive polymer demonstrate great potential in realization of versatile soft robot [1,2,3,4]. Among extant studies on electroactive polymers, the number of studies involving ionic polymer metal composites (IPMCs) has increased substantially since they were first reported two decades ago. IPMCs have been widely studied as soft actuators, actuators, and biomimetic robotics because of their desirable characteristics such as superior chemical and mechanical stability, low driving voltage, and large deformation behaviors [5,6,7].

Conventional IPMC actuators are composed of ionomeric membranes sandwiched between two metal-plated electrodes [8,9]. Commonly studied ionomers include Nafion and Flemion, for which the metal electrodes are typically composed of platinum, gold, or nickel [10,11,12,13]. Electroless chemical reduction (e.g., diffusion, absorption, and reduction) of metal salts onto an ionomeric membrane is the most common electrode fabrication process, followed by an ion exchange process, which replaces the hydrogen ions with other cation types such as sodium, lithium, and potassium for improved actuation performance [14,15].

The actuation mechanism of IPMC actuator associates with the cation transport within the ionomer. When an electric field is applied across the electrodes, the cations quickly migrate to the cathode, which causes water electro-osmosis drag and diffusion effect. A recent experiment has observed that the IPMC actuation is associated with the cation movement across the ionomer in the direction normal to electrodes by fluorescence spectroscopy [16]. Meanwhile, a significant deformation induces while the cations redistribute in the ionomer under an applied electrical field. Studies have shown the concentration, intrinsic properties, and moving directions of cations have a dominant effect on the behaviour of the IPMC actuator. Besides, the dynamic motion of IPMC could be a function of driving voltage amplitude, frequency, and device geometry, thereby adding the complication of its performance investigation [17,18,19].

To describe the mechanism of IPMC actuation, research groups have been presented several models. A micromechanical model has been presented to explain the coupled ion transport, electric field, and elastic deformation of IPMC [20]. Using a black-box linear model to discuss the dynamics of IPMC actuator has been investigated [21]. An electromechanical coupling model which associates strain to charge for IPMC actuation has been reported [22]. The dynamic characteristic of micromachined IPMC devices using a molecular-scale model has been studied [23]. Poisson-Nernst-Planck based theory has received considerable attention for modelling the actuation and sensing behaviours of IPMC [24,25,26]. The model to investigate quasi-static large deformation and electrochemistry of IPMC related to Helmholtz free energy density is recently reported [27].

A mixture theory framework for mechanical modeling of IPMC was presented [28]. The IPMC is treated as the superposition of three species: the polymer backbone, the fluid solvent, and the mobile ions. This model allows for a quantitative understanding of the different electromechanical phenomena that occur in an IPMC. The physics-based models investigating specific aspects of the IPMC actuators in order to develop a control design for practical applications are reported [29]. The model of the surface electrical potential of the IPMC sensor by incorporating the physics of the polymer membrane and the electrode is presented [30]. A multiphisics model of IPMC actuators considering the dependence of the device transduction capabilities on the environmental relative humidity is also described [31].

**Figure 1:**
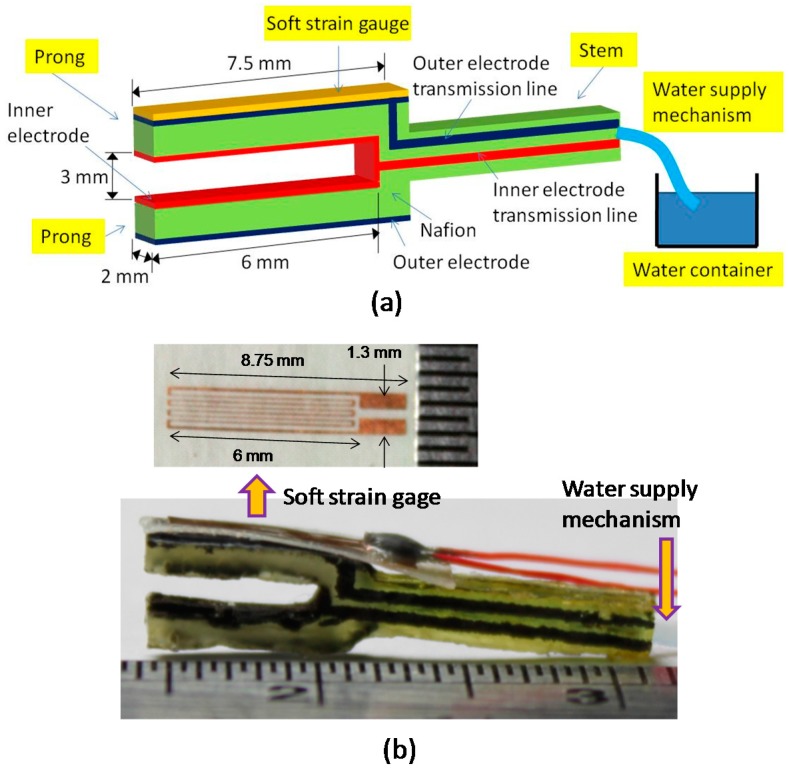
Schematic diagram (**a**) and fabricated result (**b**) of the proposed tuning-fork-shaped IPMC actuator with soft strain gauge and WSM.

A crucial parameter affecting the performance of IPMC actuation is the amount of water it contains [32]. For example, under water-saturated conditions, an IPMC actuator of a platinum electrode containing alkali cations exhibits a large relaxation deformation past its initial position toward the cathode [33]. However, once the water content decreases in the Nafion-based IPMC, the relaxation deformation also decreases [34]. We have previously developed a tuning fork-shaped clamping actuator with nickel-electroded ionic polymer metal composites [35] and found the water loss could be a crucial problem for the long-term operation of IPMC actuators in air. Recently IPMC sensors coated with a thick parylene C layer for encapsulating the entire sensor to reduce water loss were reported [36]. The experimental results showed that the coated film can effectively maintain the water content inside the IPMC. Although coating a thick parylene film could improve the rate of water loss, the IPMC actuator deposited with a thick film increases the bending rigidity and could dramatically decrease the displacement compared to a naked IPMC actuator. Our approach in this work tries to replenish the amount of water lost in the IPMC actuator with a water supply mechanism (WSM) instead of encapsulating the IPMC (Figure 1). With the proposed WSM connected to the stem (inactive) region of the actuator, the active prong of the actuator possesses the same bending rigidity as the one without WSM. Therefore, the dynamic performance of the actuator will not be degraded during long-time operation. Different from our previous work [35], two functional modules comprising a soft strain gauge and water supply mechanism are integrated in the developed tuning fork-shaped IPMC gripper actuator; systematic characterization and performance discussion are included in this work. The strain/displacement signal can be monitored in real-time from the soft strain gauge for future feedback control. The WSM injects a sufficient amount of water into the actuator to maintain the water content for continuous operation, which makes the developed actuator more practical for future commercialization. Moreover, the electrical current analysis of the developed IPMC actuator via the electrochemical model is described in detail. This gives an insight of the actuator during operation from an electrochemical perspective. Fabrication and characterization of this actuator, along with performance comparison with an identical actuator without WSM, are described in the following section.

## 2. Device Design and Fabrication

### 2.1. Design Concept

The proposed tuning fork-shaped IPMC actuator consists of two major features: two prongs serving as an active gripper and a stem enclosing a water-absorbent material for supplying water to the two prongs (Figure 1).Working electrodes were patterned separately onto the outer and inner surfaces of the prongs, enabling them to perform clamping and releasing motions under electrical control. Control signals were transmitted through the patterned electrodes on the stem to activate the prongs. The end portion of the absorbent strip outside the stem was immersed in the water-filled container to continuously feed water to the prong during operation.

Furthermore, a soft strain gauge was developed for measuring the strain of materials with relatively low Young’s moduli (*i.e.*, in the megapascal scale) [37]. The gauge was constructed using a piece of single-sided adhesive copper foil tape and a piece of Scotch tape (8884 Scotch Stretchable Tape, 3M Co., Two Harbors, MN, USA). Copper foil tape was patterned as a slender coil to enable measurement of the strain possessing the highest resistance variation and strain sensitivity in the length direction. The adhesive size of the patterned copper foil tape was utilized to secure it to the (polyethylene) back of the Scotch tape. The soft strain gauge was directly bonded to the surface of the prong by the synthetic rubber adhesive of the Scotch tape without using any extra glue. This gauge facilitated *in situ* monitoring of the strain on the surface layer of the prong.

### 2.2. Fabrication

A micromolding technique was employed to fabricate the tuning-fork-shaped IPMC actuator. The shape of the designed actuator was patterned on 1 mm thick SU8 photoresist which was coated on a glass substrate. A polydimethylsiloxane (PDMS) mold was fabricated based on the patterned SU8 die. An absorbent strip was placed inside the stem portion of PDMS mold. The absorbent strip was made from a blend of natural cotton and synthetic rayon with diameter about 1.2 mm (a product of Kotex Co., Taiwan). A sufficient amount of liquid Nafion was pipetted into the mold and then allowed to solidify at room temperature. The liquid Nafion D2021 (DuPont Co., Wilmington, DE, USA) contains 22% perfluorosulfonic acid/TFE copolymer and 78% organic solvent/water. Once the solid Nafion device was formed, a tape-made shadow mask with defined inactive openings was attached to it. AZ5214 photoresist was applied to the opening region as a selective protector during subsequently electroless chemical deposition. A 1 M nickel sulfate solution (NiSO_4_) was prepared and the shaped Nafion device was immersed in the solution for 12 h. Next, the Nafion device was rinsed with DI water and immersed in a LiBH_4_ reduction agent for nickel deposition to complete the IPMC actuator fabrication. The ion exchange process was followed by treating the IPMC devices with a 1 M sodium hydroxide solution for 12 h.

The fabrication process of the soft strain gage started with sticking a piece of copper-based conductive tape to a glass slide [37]. The tape consisted of a 22 μm-thick copper foil and a 35–40 μm thick adhesive layer (Wida Tech Printing Co., Taiwan). To form the coiled-wire pattern on this conductive tape, a standard photolithography process was performed. The wire-patterned tape was peeled from the glass and attached to the polyethylene backing layer of a diced 3M 8884 type Scotch tape. At this moment, the soft strain gauge was complete. The finished gauge was glued to the prong of the IPMC actuator by the adhesive of the Scotch tape itself (Figure 1).

## 3. Experimental Setup

The dynamic properties of the fabricated IPMC actuator with and without a WSM were characterized and compared. The actuator with a WSM was supplied with water by placing the end portion of the WSM into a water container to continuously absorb water during testing. A 5- and 7-V dc was applied to the actuator for 50 s; these two amplitudes were selected because they facilitated easy observation of significant motions in the millimeter scale. Subsequently the displacement at the free end of the prong, current flowing through the IPMC device, strain generated on the outer surface of the prong, and water volume inside the IPMC device were measured. The millimeter-range displacement allowed the pair of active prongs to open and close fully.

The millimeter-range displacement allows the pair of active prongs executes open and fully close motions. A CCD camera (Canon 500D with a frame rate of 30 fps, Canon Corp., Tokyo, Japan) was utilized in a video mode for displacement characterization. Since the feature motion of actuator is much lower than the frame rate of camera, the capture images have sufficient time resolution for analysis. An insulated clip with trimmed conductive copper tapes clamps the stem end portion of IPMC actuator for mechanical securing and transmitting actuation signals. The captured image captured with a highest quality setting uses AutoCAD software to find the coordinate value of the measuring point.

**Figure 2:**
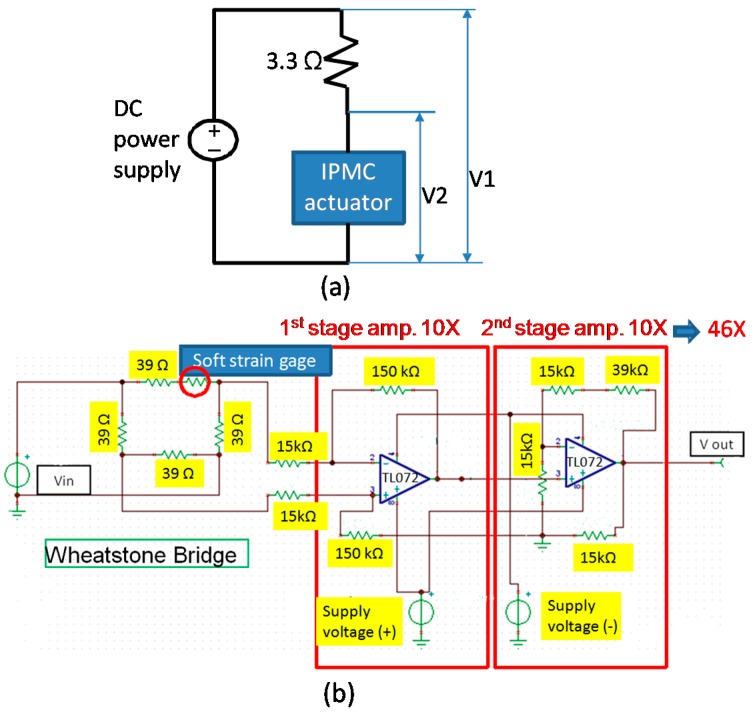
Experimental setup (**a**) for measuring the current flowing the IPMC actuator; (**b**) of Wheatstone bridge and amplifier circuitry connected to the soft strain gauge.

The current measurement employs a simple constructed circuitry (Figure 2a). An external resistor was connected between the voltage source and IPMC device. A proper resistance is selected so the ratio of the voltage across the resistor divided by the resistance R (*i.e.*, current) is in a reasonable range. In this work, a 3.3 ohm resistor was selected after several trials. A data acquisition (DAQ) system (NI-6211, manufacturer, Austin, TX, USA) is applied to obtain V_1_ and V_2_, thereby the current flowing through the IPMC actuator can be calculated as I = (V_1_ − V_2_)/R due to extremely large input impedance of the DAQ system (>1 GΩ).

The coiled wire of the soft strain gage attached to the prong of IPMC actuator was connected to a Wheatstone bridge and 46× magnifying amplifier circuitry (Figure 2b). A post anchored on a single-axis platform which has precise displacement control capability was used to push the free front end of the prong in both gripping open and close directions. This bends the prong and causes the resistance of the strain gauge to change as well as an output voltage variation through the Wheatstone bridge and amplifier circuitry. We recorded the deflection at the free end of prong and the corresponding output voltage. The generated strain at the surface of the prong for per unit output voltage change can then be characterized. For a more detailed calibration procedure readers may refer to [37].

The water content of the IPMC actuator was investigated by gravimetric analysis. The ambient temperature and humidity was about 25 °C and 50% RH. The test IPMC actuator was soaked in DI water for 4 h to allow saturation. After removal of the water and drying of the surface, the actuator was measured on an electronic balance (resolution of 0.1 mg). The mass mi is recorded when the readout reached an equilibrium state. Then we quickly started to execute the actuation of IPMC device. The mass mf was measured right after finishing the test. The IPMC actuator without WSM performs the identical experimental procedure. In addition, we dehydrated the device without WSM by heating up to 80 °C for 1 h and recorded the mass m_d_ after finishing the actuation.

## 4. Results and Discussion

### 4.1. Displacement at the IPMC Actuator Prong

**Figure 3:**
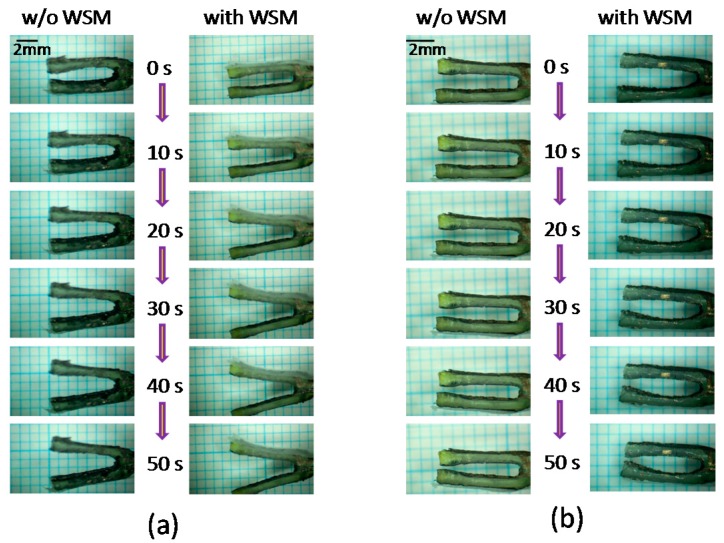
The qualitative results of driving IPMC actuator with and without WSM by 7V dc to (**a**) open state; (**b**) closed state.

Figure 3 shows the qualitative results of driving the IPMC actuator with and without the WSM by applying a 7-V dc in different states. The successive photographs reflect time intervals of 10 s. Significant open-and-close motions of the prongs were observed. Applying a 7-V dc produced larger motions compared with applying a 5-V dc.

**Figure 4:**
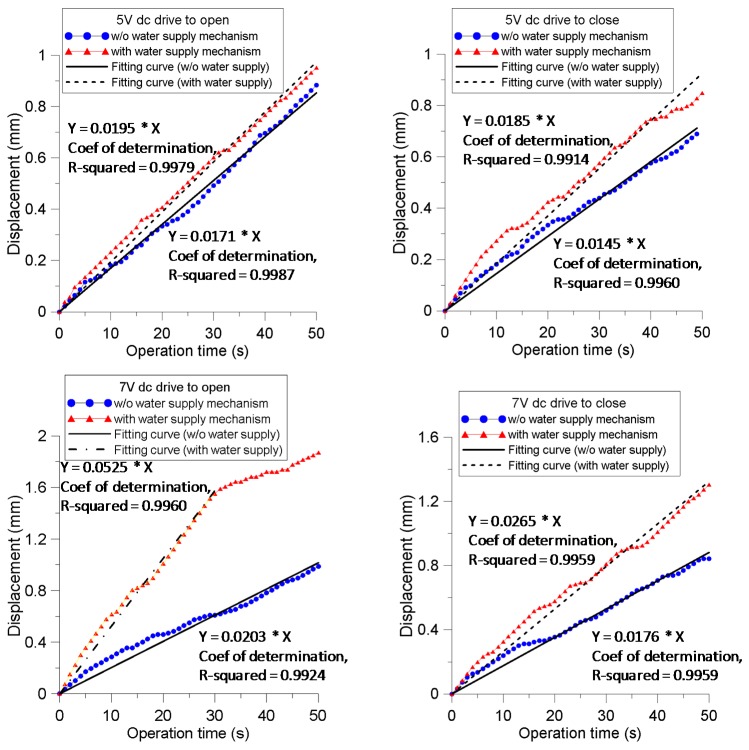
Results of displacement measured at the free end of IPMC actuator prong for different studied cases.

Figure 4 shows the measured displacements at the free ends of the IPMC actuator prongs. The displacement is measured with respect to the outer surface of the prong (front end) and with zero corresponding to the initial status. The measured direction is in the y direction. All cases revealed an increasing trend with a certain degree of fluctuation. When the 5-V was applied to drive the prongs to open, a relatively linear motion was observed. The fitting curve in the figure shows a moving speed of 17.1 and 19.5 μm/s for the cases without and with the WSM, respectively. The cases where a 5-V dc drive was applied to close the prongs of the IPMC actuator without the WSM increased the closing speed initially, before gradually reaching a relatively steady slope at approximately 25 s. The fitting curves show a closing speed of 14.5 and 18.5 μm/s for the cases without and with the WSM, respectively, both of which are lower than the corresponding values for the cases where the same actuation voltage was applied to drive the prongs open.

For the cases in which a 7-V driving force was applied to open the prongs, the actuators without and with WSM exhibited contrasting behaviors. In the case without the WSM, the displacement exhibited an approximately linear relation as actuation time increased, and the fitting curve showed a speed of 20.3 μm/s. However, the case with the WSM demonstrated a very high moving speed of 52.5 μm/s for up to 30 s, reaching a displacement of 1.5 mm, then suddenly decreasing to a near-zero speed for up to 50 s. This could be because the prong approached its maximum strain limit while the accumulation of the hydrated cations reached a saturation state in the double-layer region. The cases of 7 V driving to close the prongs revealed a nearly linear curve. After 50 s activation, the displacements reached 1.2 and 0.8 mm with respective moving speeds of 26.5 and 17.6 μm/s for the actuator with and without the WSM. This is similar to the 5V actuation cases; the case driving the prongs to open had a higher speed compared with the case driving to close, both with and without the WSM.

Considering the maximum manipulating span of the prong at free end,* i.e.*, the largest displacement when performing a closing motion plus the largest displacement while performing an opening motion, the span of the prong with WSM can reach 2 and 3.5 mm when driven with 5 and 7 V, respectively; while the span of the prong without WSM reaches 1.6 and 1.8 mm when driven with 5 and 7 V, respectively. Furthermore, the maximum separation between two prongs at the free ends could reach 7 mm from the closing to opening motion when the actuator with WSM operated at 7 V.

### 4.2. Current Response of the IPMC Actuator and Analysis via the Electrochemical Model

**Figure 5:**
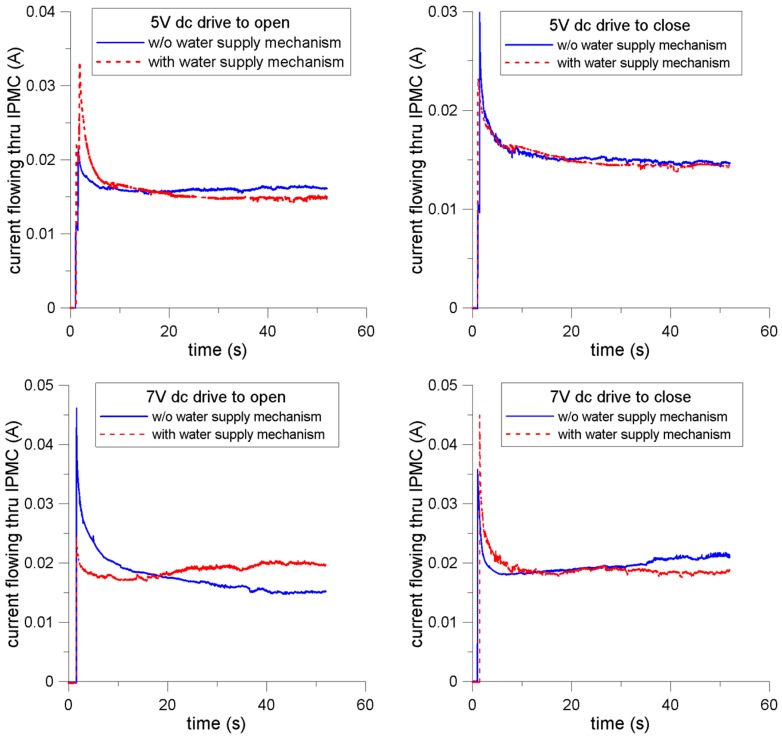
Results of the current flowing through the IPMC actuator when external voltages of 5 V and 7 V are applied to either open or close the prongs of the actuator.

Figure 5 shows the results of the current flowing through the IPMC actuator when applying voltages to operate the prongs. All the currents demonstrated a rapid upsurge, followed by a slow decreasing trend. Dissimilarity was observed between the 5 V and 7 V cases. The currents in the 5 V cases approached a steady-state after approximately 20 s. However, the currents in the two cases of 7 V driving to open the prongs (with WSM) and driving to close the prongs (without WSM) exhibited upward phenomena after reaching the minimum points, approximately 5–10 s after activating the motion. Considering the high voltage applied to the electrodes, involving a Faradaic current and high strain change rate, we believe this was because the greater contact area of the electrodes dynamically generated and reacted with water molecules, thereby increasing the charge transfer rate.

Most previous studies have described the charge configuration and current behavior of IPMC actuators by using Nernst-Planck and Poisson equations. The Nernst-Planck equation describes the migration, diffusion, and convection of charged particles inside the hydrated Nafion, whereas the Poisson equation describes the electric field formation inside the polymer. However, regarding the boundary conditions for solving the Poisson and Nernst-Planck equations, insulation conditions are often applied at the electrodes. Hence, no mass-charge transfer occurs at the interface between the electrode and ionic polymer. The non-Faradaic current gradually fades when a galvanic voltage is applied.

Nevertheless, our experimental results clearly revealed a non-zero phenomenon after a steady-state, maintaining a relatively high near-constant value. To explore the physics underlying the measured current, we used the electrochemical equivalent circuit for analysis. The heterogeneous charges were aligned at the interface; this functioned as a plate capacitor, which can be modeled as double-layer capacitance C_d_. The speed of the charge transfer was characterized by the Faradaic current. Because of the slow response of the charge transfer process, the Faradaic current induced an electrochemical polarization overpotential. The relationship between the current and potential can be represented by an equivalent resistance, namely the charge transfer resistance R_ct_. Because the voltage across R_ct_ was established by adjusting the charge state of the double layer, this voltage was equal to the voltage across the capacitance of C_d_. Hence, R_ct_ could be modeled as a parallel connection with C_d_. In addition, the ohmic potential drop when the current flowed through the electrolyte inside the Nafion can be described as R_u_ in a series connected with C_d_ (or R_ct_). Finally, while the current flowed through the interface of the electrode and electrolyte, the reactant consumption and product accumulation caused the concentration difference.

In electrochemical analysis, the diffusion impedance (Z_w_=R_w_ + 1/jωC_w_) is commonly employed to model the concentration difference effect and is connected with R_ct_ in series because the process speed of the charge transfer and diffusion mass transfer are equal, as are the Faradaic currents flowing through Z_w_ and R_ct_. However, the quantization of diffusion impedance is commonly determined through sinusoidal signal testing and is unsuitable for modeling in the case of an applied dc voltage. We thus modified the diffusion effect as a fixed resistance R_diff_ and a time-varying current source I_v_(t) to describe the variation between the measured and modeled current flowing through R_u_, R_ct_, R_diff_, and C_d_. In this study, i_v_(t) was considered similar to the reactance part of the diffusion impedance C_w_ to describe the Faradaic current perturbation resulting from the diffusion effect.

**Figure 6:**
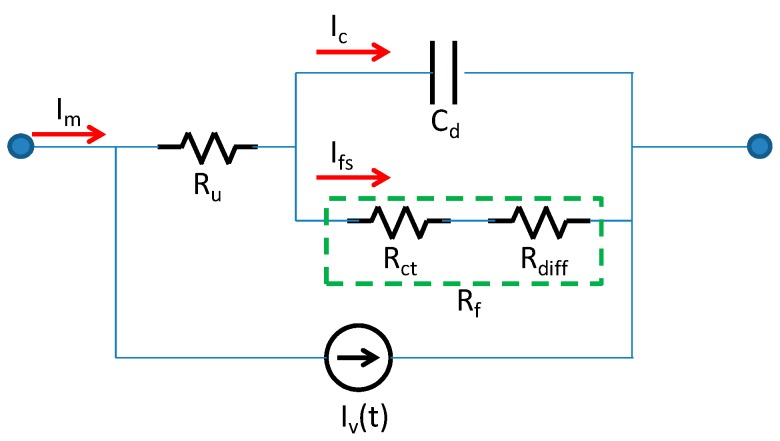
Electrochemical circuit model for analysis of measured current.

The equivalent electrochemical circuit of the fabricated IPMC actuator is shown in Figure 6,where R_u_ is the electrolyte resistance, R_ct_ is the charge transfer resistance, R_diff_ is the diffusion resistance, C_d_ is a double-layer conductance, and η is the potential across the nodes. These parameters correspond to the processes of ionic conduction (R_u_), electrical charge transfer (R_ct_), diffusion mass transfer (R_diff_), and double-layer charging (C_d_). Because R_ct_ and R_diff_ are connected in series and their established overpotential is equal to that of C_d_, we defined the Faradaic resistance as R_f_ = R_ct_ + R_diff_. Thus, the measured current (i_m_) flowing through the IPMC actuator can be modeled as non-Faradaic(i_c_) and Faradaic (i_f_) currents. The Faradaic current can be further considered as being composed of steady (i_fs_) and fluctuating i_v_(t) parts, as follows:

(1) $$i_{m}\left(t\right)=i_{c}\left(t\right)+i_{\text{fs}}+i_{v}\left(t\right)$$

Defining $i\left(t\right)=i_{m}\left(t\right)-i_{v}\left(t\right)=i_{c}\left(t\right)+i_{\text{fs}}$, we have:

(2) $$i_{c}=\frac{C_{d}\left(d\left(\eta -{\text{iR}}_{u}\right)\right)}{\text{dt}}$$

(3) $$i_{\text{fs}}=\frac{\eta -{\text{iR}}_{u}}{R_{f}}$$

(4) $$i_{\infty }=\frac{\eta }{R_{u}+R_{f}}$$

(5) $$i_{t=0}=\frac{\eta }{R_{u}}$$

where $i_{\infty }\;$represents a steady-state non-Faradaic current. We considered this value as the lowest limit of the measured current. The term $i_{t=0}$ represents the current under the initial conditions. The current subtracting the fluctuating Faradaic current from the measured current can be derived:

(6) $$\int_{i=\frac{V_{1}}{R_{u}}}^{i}\frac{d\left[i\left(R_{u}+R_{f}\right)+\eta \right]}{i\left(R_{u}+R_{f}\right)+\eta }=\frac{R_{u}+R_{f}}{R_{u}R_{f}}\frac{1}{C_{d}}\int_{0}^{t}\text{dt}$$

(7) $$i\left(t\right)=i_{\infty }\left\{1+\frac{R_{f}}{R_{u}}\exp\left[-\frac{t}{\left(R_{u}∥R_{f}\cdot C_{d}\right)}\right]\right\}$$

The time constant $\tau =\left(R_{u}∥R_{f}\right)\cdot C_{d}$ is was determined by identifying the interval between the measured current at the initial state down to the value of $0.37\times \left(i_{\infty }-i_{t=0}\right)$. Table 1 lists the calculated values of C_d_, R_u_, R_f_, and the sum of R_u_ and R_f_. The double-layer capacitances ranged from 5.82 to 34.9 mF. The resulting electrolyte resistances were between 150 and 288 Ω, having a similar scale as the Faradaic resistance (78–309 Ω). Although no specific relations among C_d_, R_u_, and R_f_ were observed in the studied cases, a clear trend was observed when R_f_ was added to R_u_, which is the total resistance with a voltage decrease of η. The total resistance showed increased with the applied voltage, regardless of whether the prongs were opening or closing, or whether the WSM was applied. This explains the three accumulated effects of the ionic conductive resistance in the Nafion electrolyte and the charge transfer resistance at the Nafion–electrode interface, as well as the increase in diffusion mass transfer resistance near the electrodes as the applied electrical field was increased.

**Figure 7:**
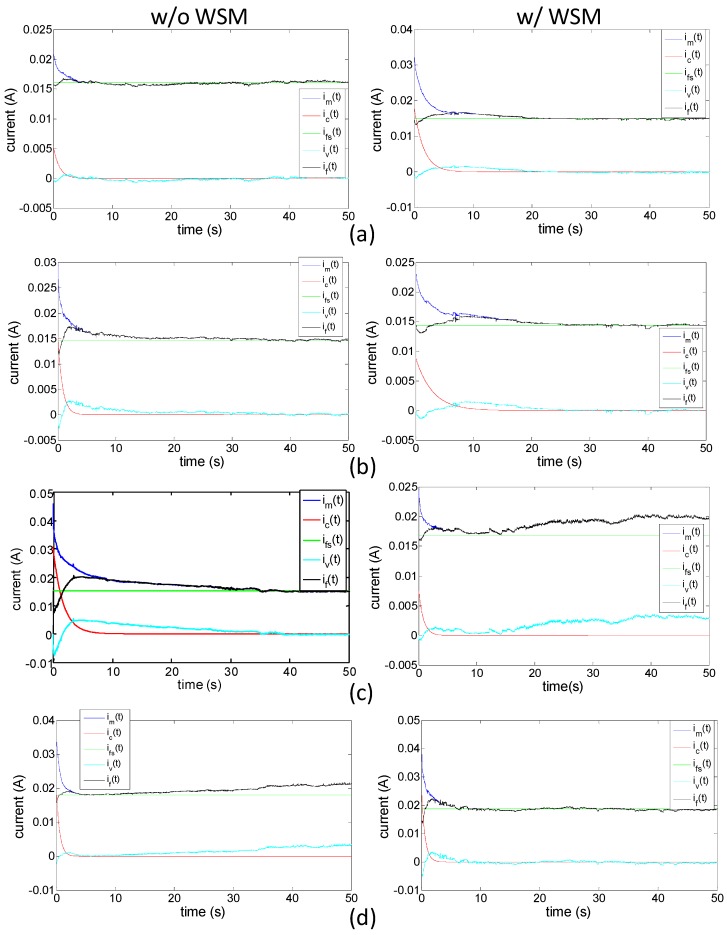
Decomposed results of the measured currents in each studied case: (**a**) 5 V driving to an open state; (**b**) 5 V driving to a close state; (**c**) 7 V driving to an open state; (**d**) 7 V driving to a close state.

Figure 7 shows the decomposed results of the measured currents. Compared with the 5-V case, the high actuation voltages produced a higher steady Faradaic current i_fs_, except in the case of when the 7-V drive was applied to open the prongs without the WSM. This is because of the markedly higher R_f_ value (308.7 Ω) in the 7-V case. While the applied voltage was high, the initially accelerated charge transfer rate at the interface generated excess products, such as oxygen or hydrogen, which gradually decreased the charge transfer rate, causing high resistance R_ct_. Moreover, the water near the double layer in the Nafion was consumed through this process. Once the water-molecule consumption rate exceeded the refill rate, the diffusion resistance R_diff_ increased. Neither effect dominated, resulting in a relatively low current.

**Figure 8:**
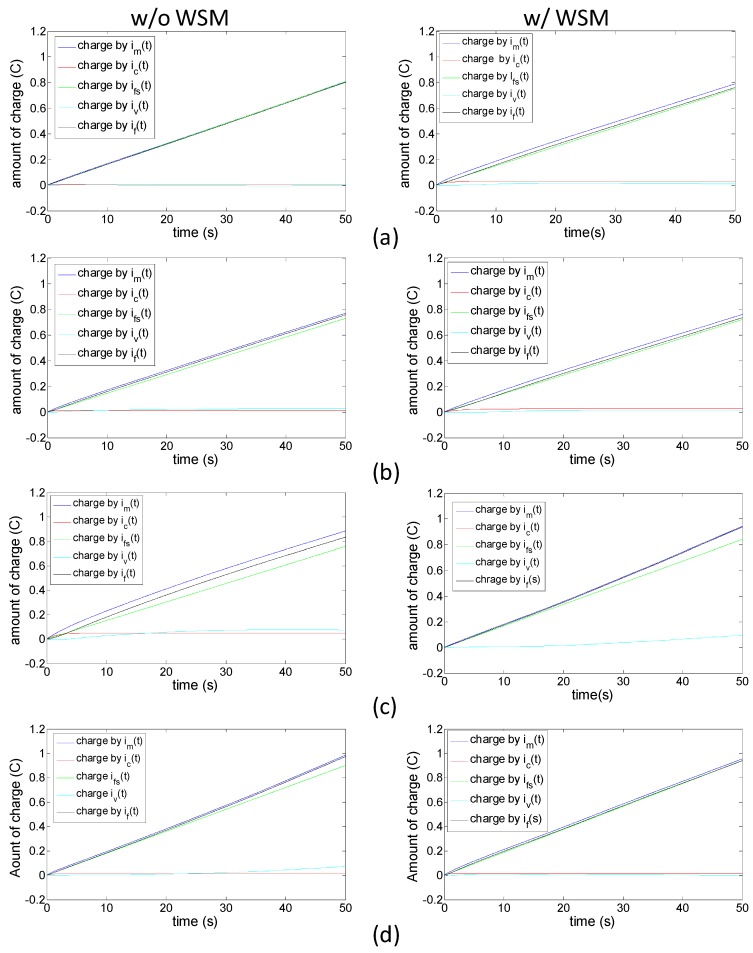
Amount of charge accumulation based on decomposed currents in each studied case: (**a**) 5 V driving to an open state; (**b**) 5 V driving to a close state; (**c**) 7 V driving to an open state; (**d**) 7 V driving to a close state.

The trend in the total charge count revealed that the high-voltage drive caused a high charge quantity, regardless of whether the prongs were opening or closing, or whether the WSM was applied (Figure 8). Moreover, the charge quantity correlated strongly with the trend of prong displacement. No clear variation in accumulated charge was observed between the actuator with or without the WSM. The case with the most notable difference (approximately 6%) was when the 7-V drive was applied to open the prongs (the value for each of the other three cases was approximately 3%). The substantial difference between the actuator with that without the WSM could be attributed to the relatively unstable faradaic current generated in the actuator with the WSM; in other words, applying a lower drive voltage produces a low charge accumulation for i_v_(t) over time.

Furthermore, the three-fold time constant for the non-Faradaic current was under 5 s, except when the 5-V drive was applied to close the prongs using the actuator with the WSM applied (the closing time was 8.6 s), possibly because its initial status differed significantly from that of the other cases. We can reasonably assume that the non-Faradaic current faded within 10 s and that it was dominant after 10 s in the studied cases.

### 4.3. Strain Relation between the Signal of the Soft Strain Gauge and Measured Displacement

**Figure 9:**
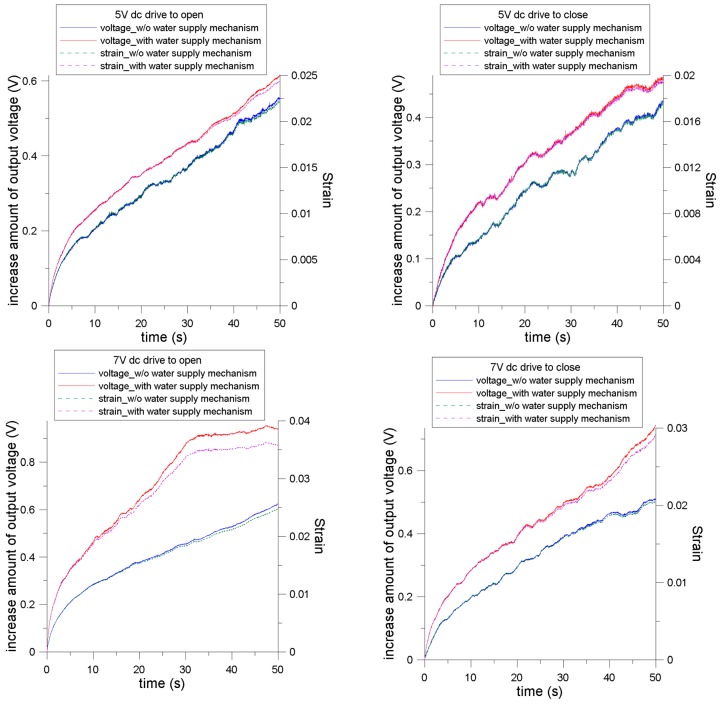
Measured electrical signal results of the soft strain gauge as a function of time and the associated strain.

Figure 9 shows the signals measured using the soft strain gauge. According to the aforementioned calibration, the measured voltage and applied deflection of the soft strain gauge showed a relation of 1.74 µm/mV. According to this relation, we determined that the corresponding deflection of the prong was subjected to a concentrated load at the free end. The corresponding strain could thus be calculated. Because of the large motion of the cantilever-like prongs, we referred to the expression for the curvature of a cantilever, denoted as κ of a cantilever.

For a cantilever of length L with deflection $\delta_{B}$ at the free end, because of the concentrated load at the same point, the deflection v at location x away from the clamped end can be expressed as:

(8) $$v=-\frac{\delta_{B}}{L^{3}}\frac{x^{2}}{2}\left(3L-x\right)$$

(9) $$\kappa =\frac{1}{\rho }=\frac{d\theta }{\text{ds}}=\frac{d(\tan^{-1}v^{\prime })}{\text{dx}}\frac{\text{dx}}{\text{ds}}$$

where, ρ is the radius of the curvature; θ is the angle of rotation; $\text{ds}={\left({\text{dx}}^{2}+{\text{dv}}^{2}\right)}^{1/2}$; and $\frac{d(\tan^{-1}v^{\prime })}{\text{dx}}=\frac{v^{\prime \prime }}{1+{\left(v^{\prime }\right)}^{2}}$.

We have:

(10) $$\kappa =\frac{1}{\rho }=\frac{v^{\prime \prime }}{{\left[1+{\left(v^{\prime }\right)}^{2}\right]}^{3/2}}=-\frac{3\delta_{B}L}{{\left(4L^{6}+9\delta_{B}{}^{2}L^{4}\right)}^{1/2}}$$

and the strain of the soft strain gauge follows $\epsilon_{x}=-\frac{y}{\rho }$, where y denotes the distance from the neutral axis to the surface of the gauge. The neutral axis can be determined by the equivalent Young’s moduli of the IPMC actuator with (13.65 MPa) and without (12 MPa) the strain gauge, which were obtained from [37]. Because the thickness and width of the actuator and strain gauge are known, the neutral axis can be calculated using the following equation [38]:

(11) $$y_{n}=\frac{\left(Y_{1}t_{1}{}^{2}+Y_{2}\left({\left(t_{1}+t_{2}\right)}^{2}-t_{1}{}^{2}\right)\right)}{2\left(Y_{1}t_{1}+Y_{2}t_{2}\right)}$$

(12) $$Y_{\text{tot}}\cdot \frac{1}{12}{\text{bt}}^{3}=Y_{1}\cdot \frac{b}{3}\left[y_{n}{}^{3}+\left(1-\frac{Y_{2}}{Y_{1}}\right){\left(t_{1}-y_{n}\right)}^{3}+\left(\frac{Y_{2}}{Y_{1}}\right){\left(t_{1}+t_{2}-y_{n}\right)}^{3}\right]$$

where$\;Y_{\text{tot}}$ is the equivalent Young’s modulus of the prong with the strain gauge; $Y_{1}\;$is the Young’s modulus of the prong of the IPMC actuator;$\;t_{1}\;$and $t_{2}\;$are the thicknesses of the prong and strain gauge, respectively; and $y_{n}$ is the distance from the neutral axis to the surface of the soft strain gauge (calculated as 0.567 mm).

The evaluated strain, which was based on the voltage measured using the strain gauge, showed a voltage deviation greater than 0.4 V. All of the strain curves initially show a similar trend of rapid surging, then gradually becoming relatively linear with different slopes. In all cases, the linear relationship persisted until approximately 0.03 (mm/mm). The actuator with the WSM exhibited larger strain compared with that without the WSM under identical drive conditions. When the strain was greater than 0.035, a saturation phenomenon as observed, which is similar to the trend of the measured displacement. This implies that the outer surface of the prong with the soft strain gauge attached reached its maximal strain limit and persisted while an external voltage was applied.

Furthermore, if we calculate the strain at the outer surface of the prong (*i.e.*, at the metal-coil strain sensing layer) by using the measured displacement data (assuming that the prong bending occurs purely because of the hydrated cations along the generated moment), it follows that:

(13) $$\epsilon_{\text{cal}}=\frac{-2\delta \cdot y}{{\left(L^{4}+4\delta^{2}L^{2}\right)}^{1/2}}$$

where δ is the measured displacement at the free end of the prong, y is the distance from the neutral axis to the surface of the gauge, and L is the length of the strain gauge.

**Figure 10:**
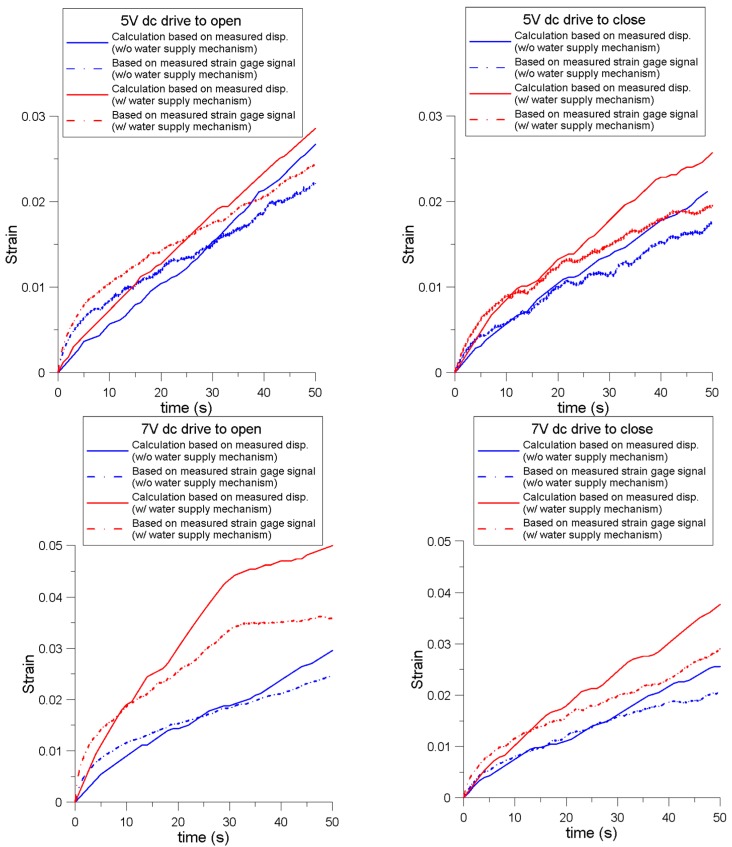
Strain comparison of the results from the strain gauge voltage signal and the results derived from the displacement of the prong subjected to a pure bending.

Figure 10 shows a comparison on the strain results derived from the strain gauge voltage signal as well as those derived from the displacement of the prong subjected to pure bending. The discrepancy was observed in all the studied cases. Initially, the strain gauge data displayed higher values compared with the estimated displacement. Beyond a certain value, this trend reversed. The cation accumulation mainly started from the double-layer region (near the electrode), producing stress and strain at this region; when the thickness of the IPMC actuator was great, it is inappropriate to consider that the produced stress only generated a moment (or bending strain) to bend the IPMC actuator prong. Thus, we propose that the effective strain effect in the developed actuator can be modified as:

(14) $$\epsilon_{\text{eff}}=\epsilon_{\text{bending}}+\epsilon_{\text{axial}}$$

The strain measured using the strain gauge is $\epsilon_{\text{eff}}$; the estimated strain by pure bending from the displacement data is $\epsilon_{\text{bending}}$. The difference between the $\epsilon_{\text{eff}}$ and $\epsilon_{\text{bending}}$ curves can be denoted as $\epsilon_{\text{axial}}$. At the initial drive stage,$\;\epsilon_{\text{axial}}\;$shows positive because of the rapid accumulation of hydrated cations at the double-layer near the strain gauge. As prong bending occurred, the axial strain gradually decreased. When the bending exceeded a certain level, the negative axial strain emerged. This may be due to part of the region inside the prong causing a shrinkage effect because of the escape of hydrated cations under the applied voltage; this effect spread caused volume shrinkage near the strain gauge area.

### 4.4. Effect on Water Content Variation of the IPMC Actuator

Operating IPMC actuators in air causes a decrease in water content because of evaporation. Furthermore, the water inside the IPMC contributes to electrolysis because of the high drive voltage. To characterize the extent of water loss, we recorded the mass of the IPMC actuator before and after actuation. The dehydrated mass of the actuator without the WSM was also recorded to enable calculating the proportion of water in the IPMC actuator. Both values deducted from the mass of soft strain gauge ($m_{\text{SG}}=4.2\text{ mg}$) are listed in Table 2. The results revealed that the mass loss for the IPMC actuator with the WSM were considerably less than that of the actuator without the WSM; thus, the WSM maintained the water content in the IPMC. Therefore, this could be a solution for long-term operation of IPMC actuators in air.

The Young’s modulus due to the change of water content in IPMC actuator requires further investigation. According to [39], the Young’s modulus of an IPMC actuator can be obtained using the following equation:

(15) $$Y_{\text{IPMC}}=\frac{Y_{M}Y_{N}}{{\text{BA}}_{B}Y_{M}+\left(1-{\text{BA}}_{B}\right)Y_{N}}$$

where $Y_{M}$ and $Y_{N}$ represent the respective Young’s modulus of the metal electrode and Nafionwith hydration $\bar{w}$ when the hydration of the IPMC is w); and $A_{B}$ is the concentration factor (given as 0.5) [29]. Parameter B is defined as:

(16) $$B=\frac{\left(1+\bar{w}\right)\left(1-f_{M}\right)}{1+\bar{w}\left(1-f_{M}\right)}$$

(17) $$\bar{w}=\frac{w}{1-f_{M}}$$

where w is the ratio of the volume of water absorbed divided by the dry volume of the IPMC, and $f_{M}$ is the volume fraction of the metal electrode in a dry IPMC device, which is given by:

(18) $$f_{M}=\frac{\left(1-\text{SF}\right)\rho_{N}}{\left(1-\text{SF}\right)\rho_{N}+\text{SF}\cdot \rho_{M}}$$

where SF is the scaling factor, denoting the weight fraction of the dry polymer in the IPMC; $\rho_{N}=2.01\;g/{\text{cm}}^{3}$ is the mass density of the Nafion; $\rho_{M}=8.91\;g/{\text{cm}}^{3}$ is the mass density of nickel. Thus, $f_{M}$ can be evaluated as 0.0144 in our fabricated actuator. According to Table 2, $\;\bar{w}\;$ranges ranged from 0.442 to 0.403, corresponding to the initial status (zero water loss) to the maximal water loss (2.3 mg). Consequently, the increase in B is only from 0.9896 to 0.9897, indicating that $Y_{\text{IPMC}}\;$only depends only on $Y_{N}$($Y_{M}=200\text{ GPa}$ is a constant).Meanwhile, $Y_{N}\;$ exhibited a very rapid decreasing trend in hydration percentage (<10%) and a near constant trend while the hydration percentage was over 30% [29]. We thus reasonably assumed that the IPMC actuator had an approximately identical Young’s modulus in all cases.

Furthermore, the water consumption in the hydrolysis process during actuation can be evaluated. The chemical reaction of hydrolysis is expressed as follows:

6H_2_O (l) O_2_ (g) + 4H_3_O^+^ (aq) + 4e^−^ (to anode) (at anode) (19)

4e^−^ (from cathode) + 4H_2_O (l) 2H_2_ (g) + 4OH^−^ (aq) (at cathode) (20)

The amount of water consumption can be computed according to the current. Ideally, one Faraday (96,485 C) delivers one mole of electrons, which produces 0.5 mol of H_2_and 0.25 mol of O_2_. Thus, 1 C produces 5.18 µmol H_2_ (10.455 µg) and 2.59 µmol O_2_ (82.888 µg). Therefore, 93.343 µg of H_2_O was consumed. The largest accumulated charge caused by the Faradaic current was 0.95 C. The maximum water consumption from electrolysis was thus less than 0.09 mg, indicating that the major cause of water loss was evaporation rather than electrolysis; furthermore, the WSM provided adequate water to the IPMC actuator.

## 5. Conclusions

In this study, a tuning fork-shaped IPMC actuator was fabricated and characterized. By driving the actuator with a 7-V dc, the free end of the 6 mm-long prong of IPMC actuator reached a speed of 52.5 μm/s. The actuator with the WSM exhibited greater displacement than did the one without the WSM under identical actuation conditions. The measured currents were analyzed using the electrochemical model. The results revealed that the Faradaic current dominated the operation of the IPMC actuator after 10 s. The electrolyte and Faradaic resistances indicate the dependence of the applied voltage, which increased with the applied voltage. The high drive voltage caused considerable charge accumulation, regardless of whether the actuator was opening or closing the prong, or whether the WSM was applied. The maximum strain of 0.035 could be maintained at the outer surface of the actuator. Both axial and bending strains were generated within the prong during operation. The initially positive axial strain subsequently switched to a negative strain. No clear difference was observed in Young’s moduli because the actuator with or without the WSM. Most of the actuator water loss during operation was attributable to evaporation rather than hydrolysis. Thus, the WSM effectively maintained the water content inside the IPMC actuator.

## Figures and Tables

**Table 1:** Calculated parameters of double-layer conductance, electrolyte resistance, Faradaic resistance under varying actuation conditions.

Parameters	5 V Open		5 V Close		7 V Open		7 V Close	
	w/o	w/	w/o	w/	w/o	w/	w/o	w/
C_d_ (mF)	17.63	19.1	9.19	34.9	16.0	9.11	5.82	6.38
R_u_(ohm)	232.6	150.2	167.2	214.6	151.8	288.1	195.5	155.9
R_f_ (ohm)	78.0	183.2	175.3	132.6	308.7	128.6	193.4	216.4
R_u_+R_f_(ohm)	310.6	333.3	342.5	347.2	460.5	416.7	388.9	372.3

**Table 2:** Measured masses of IPMC actuators before and after actuations.

Measured Mass (mg)	5 V Open		5 V Close		7 V Open		7 V Close	
	w/o	w/	w/o	w/	w/o	w/	w/o	w/
m_i_-m_SG_	83.7	285.5	83.9	285.8	83.8	285.4	83.9	285.6
m_f_-m_SG_	82.3	285.4	82.4	285.6	81.5	285.3	81.8	285.5
loss of mass (m_i_-m_f_)	1.4	0.1	1.5	0.2	2.3	0.1	2.1	0.1
dehydrated m_d_	58.2	X	58.7	X	58.6	X	58.9	X
